# Incidence and radiological pattern of eosinophilic granuloma: a retrospective study in a Chinese tertiary hospital

**DOI:** 10.1186/s13018-019-1158-1

**Published:** 2019-05-09

**Authors:** Sha-Sha Zhao, Lin-Feng Yan, Xiu-Long Feng, Pang Du, Bao-Ying Chen, Wen-Ting Dong, Yi Gao, Jie-Bing He, Guang-Bin Cui, Wen Wang

**Affiliations:** 1 Department of Radiology and Functional and Molecular Imaging Key Lab of Shaanxi Province, Tangdu Hospital, Fourth Military Medical University (Air Force Medical University), 569 Xinsi Road, Xi’an, 710038 Shaanxi People’s Republic of China; 2 Department of Medical Information, Tangdu Hospital, Fourth Military Medical University (Air Force Medical University), 569 Xinsi Road, Xi’an, 710038 Shaanxi People’s Republic of China; 3 Student Brigade, Fourth Military Medical University (Air Force Medical University), Xi’an, 710032 Shaanxi People’s Republic of China

**Keywords:** Eosinophilic granuloma, Incidence, Radiology, Chinese

## Abstract

**Background:**

The incidence and radiological patterns of eosinophilic granuloma (EG) in China is not clear. We described the incidence, presentation, and imaging characteristics of Chinese EG patients in a tertiary hospital.

**Methods:**

A retrospective chart review was performed from January 2004 to October 2017 at a single tertiary general hospital. Seventy-six patients were pathologically identified as EG. Besides, 60 patients with preoperative imaging diagnosis of “EG” were analyzed to reveal the radiological patterns and their diagnostic power.

**Results:**

Fifty-three male and 23 female EG patients with a mean age of 18.1 ± 16.7 years (range 1–58 years) were retrospectively included. Significant differences were observed in gender (male to female = 2.3:1) and age (the highest incidence at the age of 0~5 years) for EG. EG predominantly involved the skeletal system: flat bones (31.43%) > irregular bones (24.76%) > long bones (22.86%) > other organs (20.95%). No obvious relationships between season, biochemical markers, and EG incidence were observed. The common presenting symptoms were pain followed with local mass, and most patients underwent surgical resection. Among 60 imagingly diagnosed “EG” patients from April 2009 to October 2017, only 22 were with histological confirmation. The correct diagnosis rates were 37.1% (13 out of 35), 16.7% (5 out of 30), and 22.2% (8 out of 36) for plain radiography, computed tomography (CT), and magnetic resonance imaging (MRI), respectively.

**Conclusions:**

Chinese EG has a varied presentation, age distribution, and gender difference. EG diagnosis is still based on biopsy or histopathology instead of imaging techniques.

**Electronic supplementary material:**

The online version of this article (10.1186/s13018-019-1158-1) contains supplementary material, which is available to authorized users.

## Background

Langerhans cell histiocytosis (LCH) represents a series of diseases caused by the abnormal proliferation and tissue accumulation of dendritic cells with features similar to epidermal Langerhans cells in various organs [1]. As one of the most benign and predominant condition, eosinophilic granuloma (EG) is featured by a clonal proliferation of Langerhans-type cells with a solitary osseous lesion, accounting for 60 to 80% of LCH individuals [2, 3]. In the clinical settings, not all EG need to be treated with surgery, because EG are frequently with potential spontaneous remission [4]. Previous studies revealed several epidemiological features of EG, including a peak incidence at 1–5 years, a male predominance (male/female ratio, 3.7/1), and a seasonal prevalence [5]. However, these data were obtained mainly from western countries; the incidence and radiological patterns of EG in the Chinese population remain unclear.

Besides the unclear epidemiological features of Chinese EG patients, the preoperative imaging diagnosis of EG remains unsatisfying. Commonly, the classic radiographic characteristics are utilized for the basic assessment of osseous lesions [6]. Plain radiography offers the full range view of the lesion, and computed tomography (CT) is for determining the characteristics and extent of bone lesions. Magnetic resonance imaging (MRI) is ideal for detecting an abnormal signal intensity and has been utilized to make a more accurate diagnosis [7–9]. However, a definitive diagnosis of LCH should always be based on histological and immunohistochemical examinations of lesion tissue, which is characterized by abundant eosinophilic cytoplasm and positive CD1a and/or CD207 (Langerin) staining of lesion cells [2, 10]. Preoperative imaging diagnosis of EG, although very important for optimizing therapeutic strategy, is still far from satisfying in the clinical settings.

Thus, it is very important to reveal the incidence and radiological patterns of EG so as to aid accurate diagnosis in Chinese patients. We performed the current retrospective study to assess the incidence of pathologically confirmed EG in a tertiary hospital from January 2004 to October 2017 and described the radiological patterns of different imaging modalities. We also investigated the skeletal involvement at diagnosis and clinical findings in this patient cohort.

## Methods

### Patients and methods

This retrospective study was approved by the institutional review board of Tangdu Hospital of the Fourth Military Medical University, and informed consents were waived from all participants by the ethics committee of Tangdu Hospital. A retrospective chart review was performed from a pre-existing clinical database which contains all patients with imagingly or pathologically confirmed EG lesions from January 2004 to October 2017. Only patients with pathologically confirmed EG were included for incidence and radiological pattern analyses. Imagingly diagnosed “EG” with the confirmative pathological diagnosis (not always as EG) was included to analyze diagnostic powers of different radiological modalities (plain radiography, CT, or MRI). The charts were reviewed for demographic data, presenting symptoms and pathologic findings. Three radiologists (ZSS, YLF, and DWT) reviewed all images. Plain radiography, CT, and MRI images were reviewed with attention to location, laterality, and multifocality and to evaluate osseous lesions and involvement of soft tissues.

### Statistical analysis

Continuous variables were reported as mean ± standard deviations (SD). Categorical variables were summarized with frequency counts and percentages. Statistical graphs were produced with GraphPad Prism 6.01 (GraphPad Inc., La Jolla, USA).

## Results

### Completeness and quality of data

**Fig. 1:**
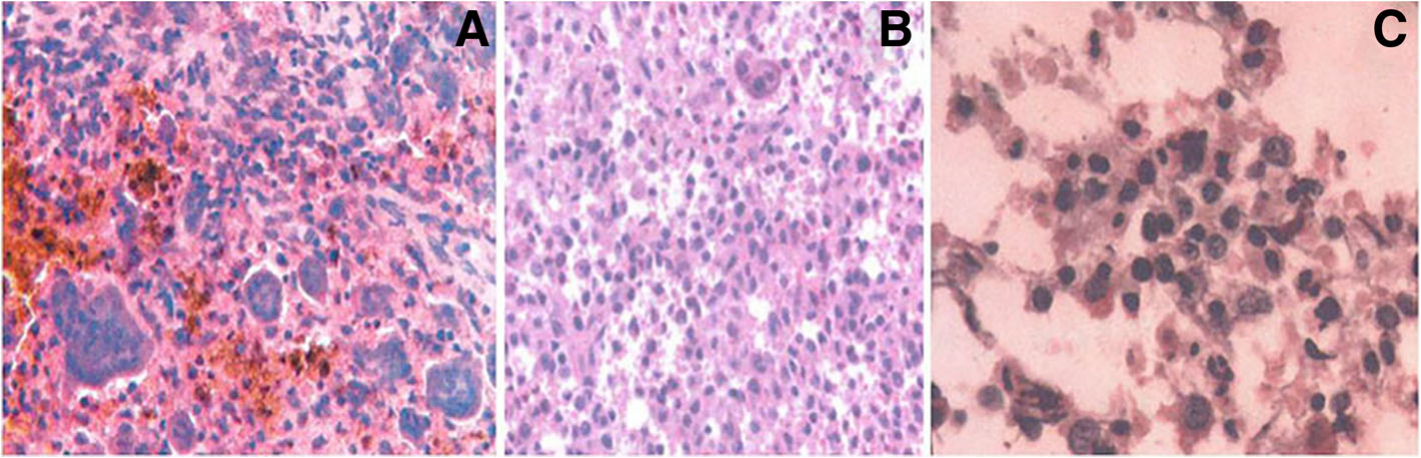
**a**, **b** The tumor tissue were composed of oval or round mononuclear cells and mixed osteoclast like multinucleated giant cells, with patchy hemorrhage, a few scattered mononuclear cells, and eosinophilic infiltration. **c** Bone marrow biopsy can be useful in the diagnosis of eosinophilic granuloma (EG). The granulocytic proliferation was active and can be seen in every stage. The erythroid hyperplasia was active, and red blood cell family can be seen. There were many megakaryocytes

Between 2004 and 2017, 76 confirmed EG cases were retrieved from our hospital and 60 imagingly diagnosed “EG” cases (22 were pathologically confirmed as EG) were retrieved from 2009 to 2017 when the image data were available. The EG diagnosis was all made based on the histology analyses of open biopsy from 64 patients, and fine needle aspiration (FNA) from the remained 12 patients (Fig. 1). For all cases, the information was sufficient to code the EG as unifocal or multifocal lesions (Additional file 1: Table S1).

### Estimation of EG incidence based on the hospital database

Because this study was conducted on a single hospital where the patients were not limited to a specific geographic region, we could not obtain the population-based incidence. Thus, it should be noted that the “incidence” used in the current study is different from the conventional ones. However, we could indeed get the age, gender, and season distributions of EG cases.

The overall incidence of EG fluctuated during the reviewed 14 years, with two peaks in 2007 and 2011, two valleys in 2004 and 2008, and two gradual increasing tendency during 2004 to 2007 and 2008 to 2011. Besides, the male incidences were always higher than those in the female population, and the ratio of male to female is 2.3:1 (Additional file 2: Figure S1). Twenty-five (7 female), 12 (5 female), 5 (1 female), 5 (1 female), 8 (2 female), 3 (1 female), 4 (1 female), 4 (1 female), and 5 (4 female) cases occurred at the age of 0–5, 6–10, 11–15, 16–20, 21–25, 26–30, 31–35, 36–40, and 51–55 years, respectively (Additional file 2: Figure S1). Again, the male predominance of EG existed for almost all age populations.

The seasonal variation of EG incidence is reported in a previous study performed in north European population [5]. We also revealed the potential seasonal variations of EG at diagnosis (Additional file 3: Figure S2). In the current patient cohort, 49 patients (64%) were diagnosed during Spring (March–May, *n* = 24) and Summer (June–August, *n* = 25), compared with 27 patients during Fall (September–November, *n* = 12) and Winter (December–February, *n* = 15). Besides, nine of 13 patients with multiple lesions were diagnosed during Spring–Summer. Forty of the 76 patients (53%) who initially presented with a single lesion were also diagnosed during the Spring–Summer period. EG may be associated with a diagnostic delay. The diagnostic delay may be from onset of symptoms, treatment delay, socioeconomic conditions, and the time of pathological diagnosis and so on.

### Primary lesion locations

**Fig. 2:**
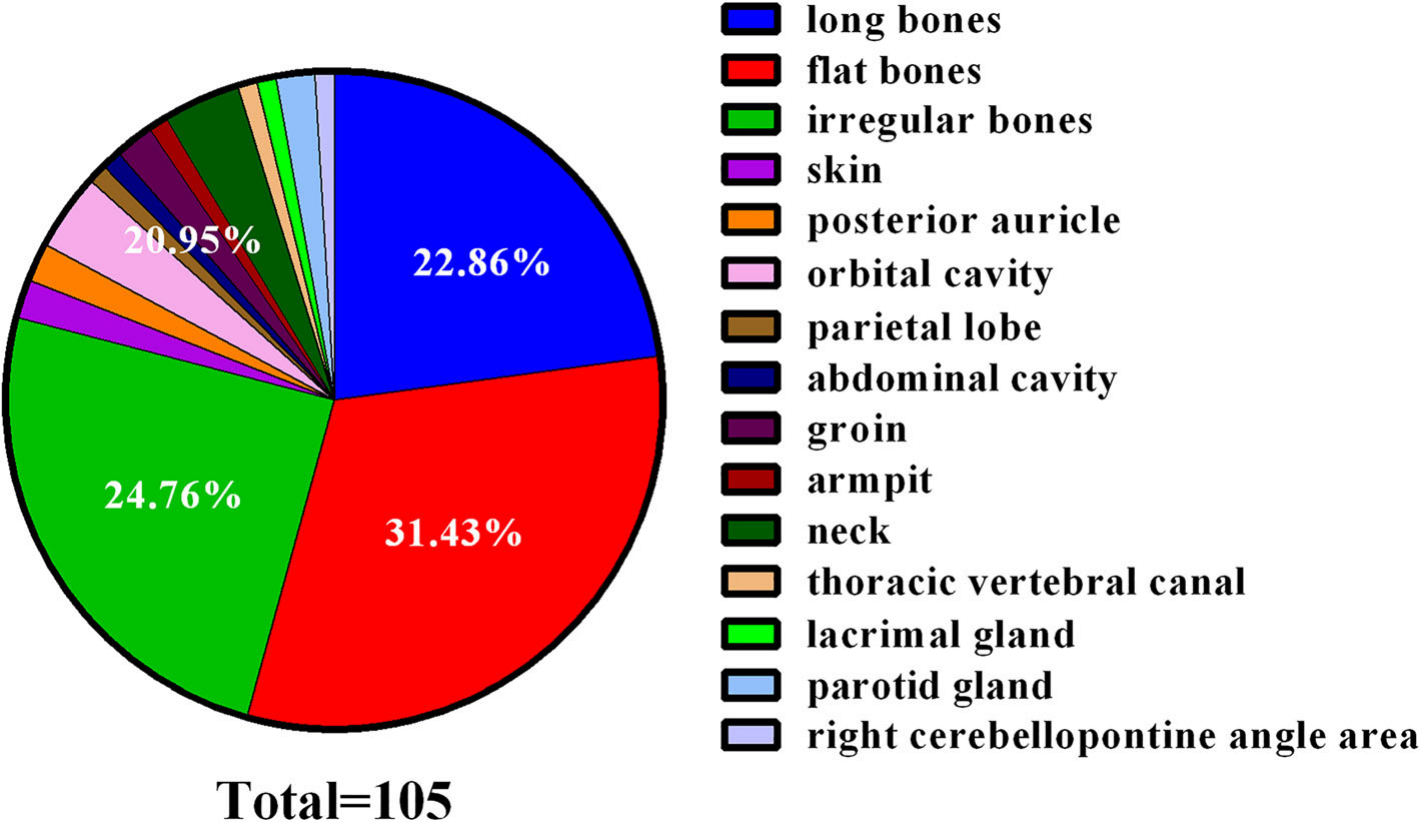
The distribution of EG (105 lesions from 76 patients)

The distribution of 105 lesions from 76 patients was shown in Additional file 1: Table S1 and Fig. 2. Overall, EG mainly affected bones (79.0%) and, to a lesser extent, skin (1.9%). The EG lesions were presented in 82.9% of the unifocal forms and 17.1% of the multifocal forms. EG predominantly involved the skeletal system according to the following order: flat bones (31.43%) > irregular bones (24.76%) > long bones (22.86%) > other organs (20.95%) (Fig. 2); the other organs here referred to skin, posterior auricle, orbit, parietal lobe, abdomen, groin, armpit, neck, thoracic vertebral canal, lacrimal gland, parotid gland, and cerebellopontine angle area.

### Radiological findings

In general, EG patients were found to be with significant radiological features including bone destruction, cortical changes, periosteal reaction or ossification, bone marrow edema, soft tissue swelling, and mass.

**Fig. 3:**
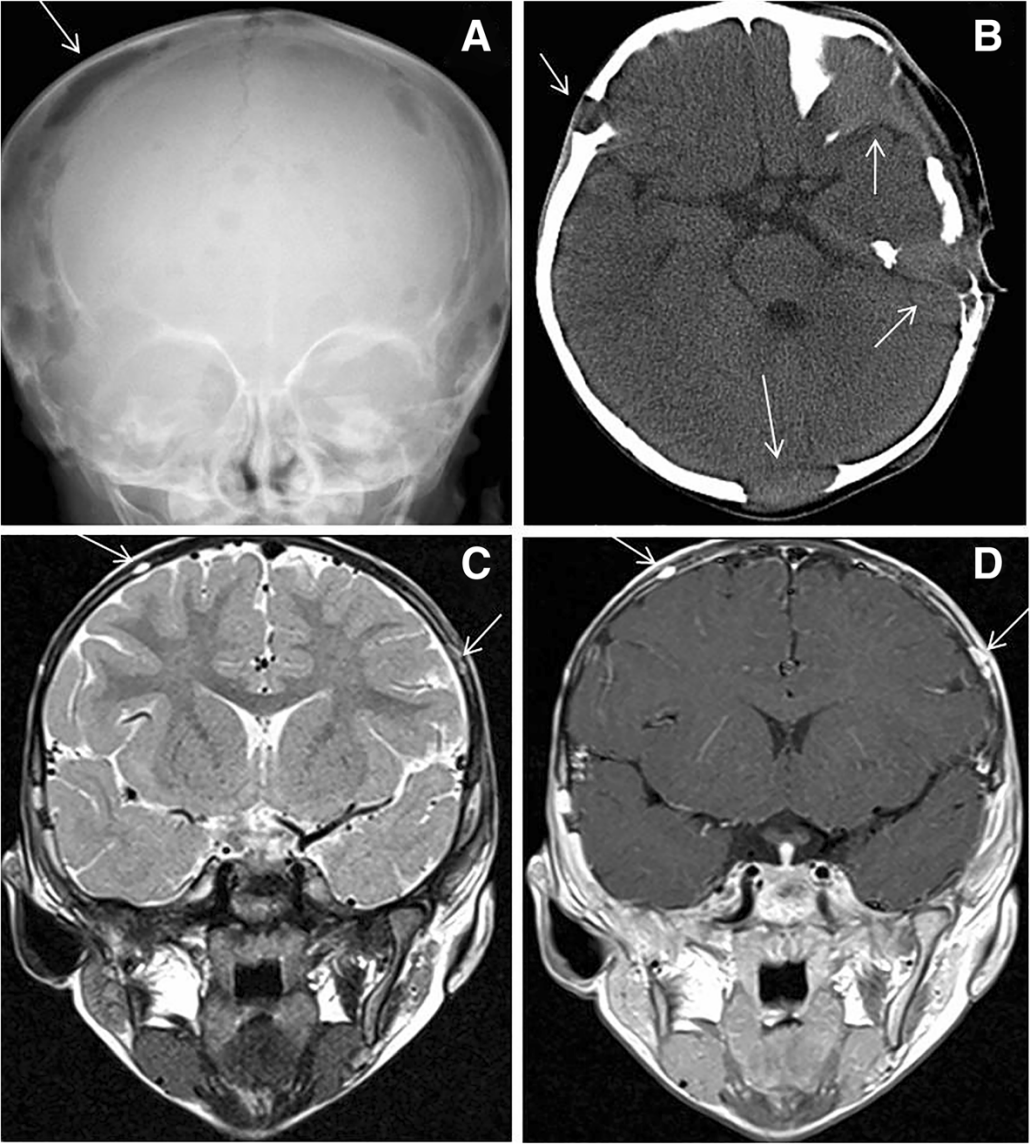
Three-year-old female presenting with multiple tumors of the skull. **a** In the plain radiography, map like bone destruction (arrow). **b** Axial post-contrast computed tomography (CT) in bone window showed an aggressive lytic lesion with no peripheral sclerosis (arrow). **c** T2-weighted high signal is demonstrated in bilateral parietal bone (arrow). **d** Axial enhanced-contrast T1-weighted MRI showed obvious enhancement (arrow)

Eight cases of skull EG were confirmed with pathology, with 3 involving frontal bone, 2 involving occipital bone, and 3 involving parietal bone. On plain radiography and CT imaging, perforated or osteolytic bone destruction, clear border, regular or irregular morphology, and very few marginal hardening were also manifested. In the skull, the lesions developed in the diploic space are lytic, and their edges may be beveled, scalloped, or confluent, or show the “buttons like” sequestrum, or the “beveled edge” with the soft tissue mass [9, 11]. In 3 EG cases diagnosed with MRI, low T1WI signal and high T2WI signal were revealed and intracranial masses were also revealed with local dura invagination (Fig. 3).

**Fig. 4:**
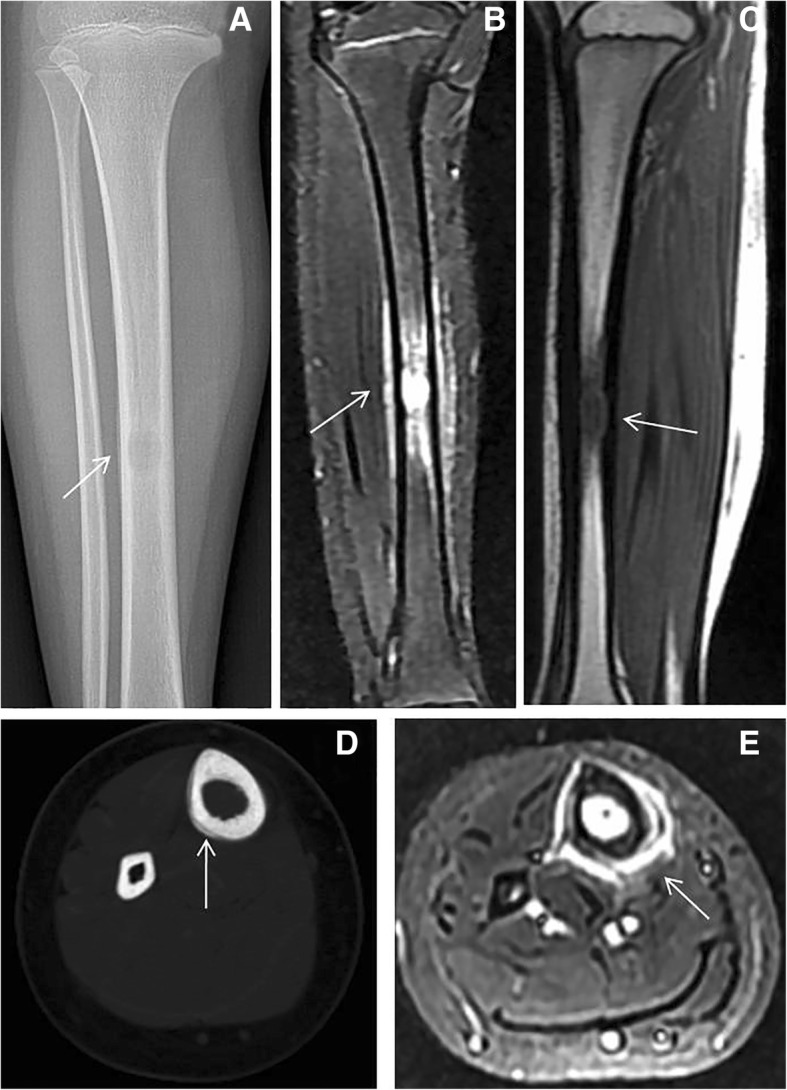
Nine-year-old female presenting with skull mass. **a**, **d** Right femur showed round cystic expansion damage surrounded with hyperplasia hardening and the layered periosteal reaction (arrow). **b**, **c** The lesion showed osteolytic destruction, T1WI showed slightly mixed lower signal, and STIR showed mixed high signal with high STIR signal in adjacent medullary cavity (arrow). **e** The image characterized osteolytic bone destruction and edema of surrounding soft tissue (arrow)

Thirty-three cases of long bone lesions, including 1 with multiple lesions, were confirmed with pathology, involving femur, humerus, ulna, and tibia. The main appearances of the plain radiography and CT imaging were expansive bone destruction, thin cortex, reactive sclerosis or periosteal thickening [12], and layered periosteal reaction. However, the periosteal reaction was different from that of malignant bone tumors. It was natural and continuous, and the layers were more parallel and well-proportioned, often in a range larger than the extent of bone destruction [11, 13–15]. Osteolytic destruction and soft tissue mass were also revealed in one MRI examination. The lesion was with slightly mixed low T1WI signal, mixed high T2WI and STIR signals, and high STIR signal in the adjacent medullary cavity (Fig. 4).

**Fig. 5:**
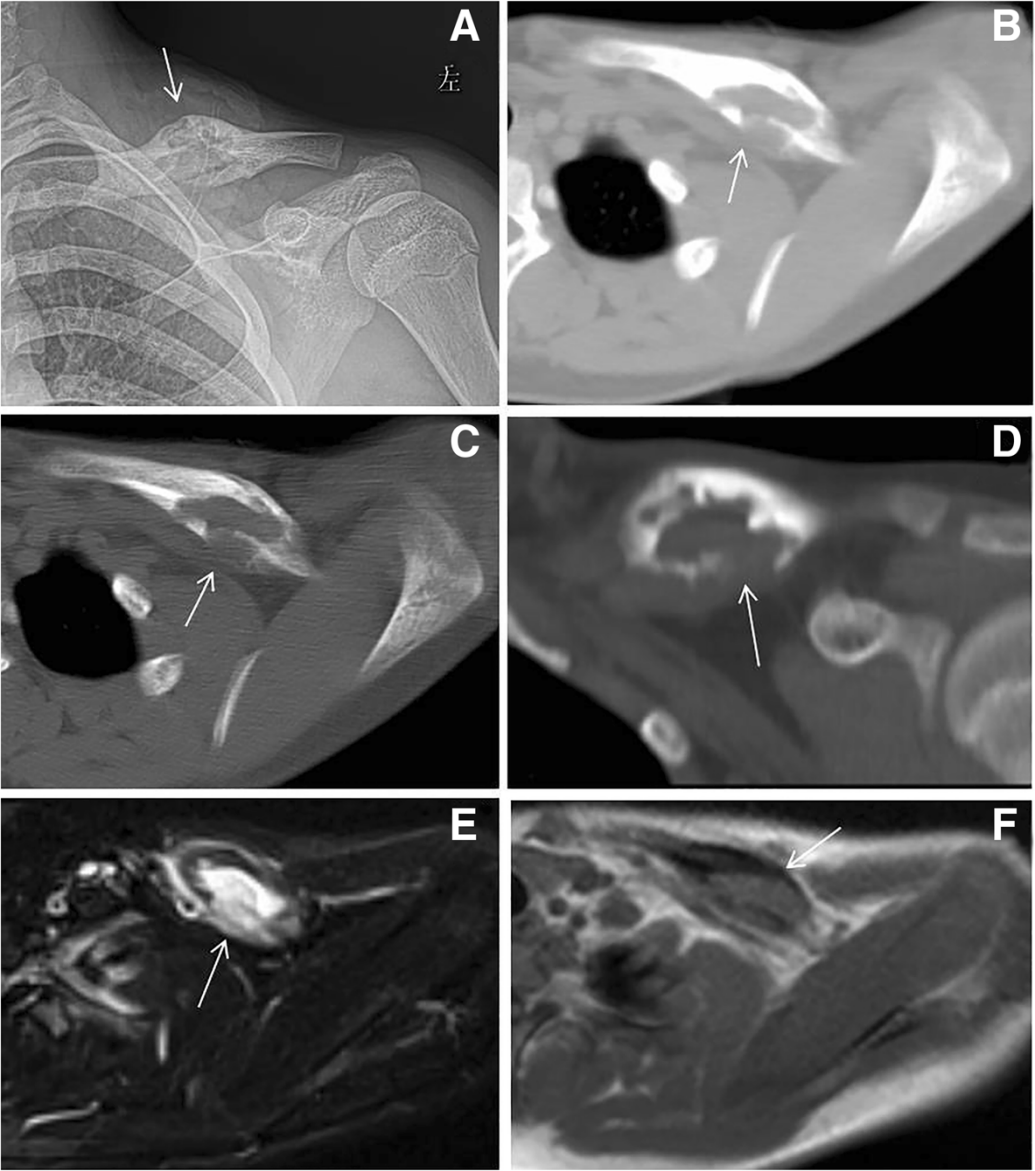
Fourteen-year-old male presenting with left clavicular lesion. **a** Expansive bone destruction on the left clavicle (arrow). **b** There was a destructive mass with a prominent soft tissue component (arrow) demonstrated on axial non-contrast computed tomography (CT) in soft tissue window. **c**, **d** Axial and coronal CT in bone window showed hyperosteogeny and sclerosis (arrow). **e** The mass had components that was hyperintense (arrow) to region on axial STIR-weighted magnetic resonance imaging (MRI). **f** Axial MRI T1 sequence revealed soft tissue mass (arrow)

The EG lesions were also revealed in iliac (5 cases), sacroiliac joint (1 case), and clavicle (3 cases) regions. These lesions were characterized as osteolytic bone destruction, sclerosis, and soft tissue mass, but without any obvious periosteal reaction (Fig. 5).

Two cases of spinal EG were confirmed with pathology, including 1 case involving the thoracic spine and 1 case involving the lumbar spine. The vertebras were flattened or wedge-shaped, called “flat vertebra” or “copper plate vertebra.” The flat vertebra is a characteristic imaging manifestation of EG [7, 13].

### Diagnostic power of varied imaging modalities for EG

A total of 60 imagingly diagnosed “EG” patients from 2009 to 2017 were retrieved, and the general information of these patients was listed in Additional file 4: Table S2. Among these 60 imagingly diagnosed “EG” patients, only 22 were histologically confirmed as EG. The correct diagnosis rates were 37.1% (13 out of 35), 16.7% (5 out of 30), and 22.2% (8 out of 36) for pain radiography, CT, and MRI, respectively. Taking together, pain radiography, CT, and MRI did not show enough power for preoperative diagnosis of EG.

## Discussion

In the current study, we retrospectively investigated 76 EG patients. The demographics and presenting symptoms of this cohort are in accordance with the published literature and reflect the wide variety of possible clinical scenarios. Overall, the disease showed a propensity for young children, male population, and seasonal variations [5, 16, 17]. Besides, the plain radiography, CT, or MRI did not offer enough power for preoperative diagnosis of EG.

Although we could not retrieve the real incidence for EG, we did reveal that EG favors young children, male population, and Spring and Summer seasons [17–19]. Because of the unique features of the Chinese medical care system, patients are not restricted to a regional hospital and the medical registry is not well established; it is not easy to get the population-based incidence. Besides, because of Chinese culture, EG patients without symptoms or with mild symptoms, such as psoriasis and gastric ulcer, may not be hospitalized to get the confirmative diagnosis. Our hospital-based incidence could have been underestimated. However, the prevalence in young children, male population, and Spring and Summer seasons is consistent with previous publications in different ethnics [20, 21]. The variable incidence is not easily understood but may be affected by several factors, including genetic and immune response factors.

Consistent with previous reports [5, 22], a possible seasonal variation of EG, with a higher incidence during the Spring–Summer was revealed in the current study. However, there is still a report that no significant incidence variation over the year was revealed for EG [23]. In a previous study among the Chinese population, the EG incidence increased during 1997–1998 in Taiwan province, with most EG cases diagnosed in the Summer when rainfall peaked [24]. The seasonal features of patients in our hospital are different from that of South China, and other regions of China since the Summer and Spring of Northwest China are relatively hot and dry without too much rain, and there have been few reports in the past [17, 24–28]. The detailed reasons for the seasonal EG incidence variations should be further investigated. Previous studies mainly focused on children and few adults in South China, Southeast China (mainly in Taiwan), and Northeast China. However, the cases included in the current study were not only children but also adults, mainly in Northwest China.

Although this retrospective study was with a small sample size and the involvement of a single institution, we feel that the review of incidence and radiological patterns of this rare disease in the modern era is a valuable addition to the literature.

## Conclusions

Our study suggested that the overall incidence of EG was not high. The histopathology of the lesions occurring in different parts and at different stages is basically similar, but the radiological characteristics vary greatly in different sites, ages, number of lesions, and stages. It is easy to cause misdiagnosis by simply relying on images and ignoring clinical data. Besides, none of the currently available radiological modalities could reach satisfying diagnostic power. Therefore, correct diagnosis is dependent on epidemiological, clinical, and imaging performance. Compositive and comprehensive analyses can improve the diagnosis accuracy.

## Additional files

Additional file 1: **Table S1.** Basic information of 76 patients. (DOCX 29 kb)

Additional file 2: **Figure S1.** Gender differences among different age groups. (JPG 293 kb)

Additional file 3: **Figure S2.** Seasonal variation of EG. (JPG 317 kb)

Additional file 4: **Table S2.** Pathological findings obtained by imaging diagnosed as “EG”. (DOCX 30 kb)

## Abbreviations

EG: Eosinophilic granuloma
CT: Computed tomography
MRI: Magnetic resonance imaging
LCH: Langerhans cell histiocytosis

## References

[1] Mitchell JM, Berzins SP, Kannourakis G (2018). A potentially important role for T cells and regulatory T cells in Langerhans cell histiocytosis. Clin Immunol..

[2] Haupt R, Minkov M, Astigarraga I (2013). Langerhans cell histiocytosis (LCH): guidelines for diagnosis, clinical work-up, and treatment for patients till the age of 18 years. Pediatr Blood Cancer..

[3] Zheng W, Wu J, Wu Z, Xiao J (2014). Atlantoaxial instability secondary to eosinophilic granuloma of the axis in adults: long-term follow-up in six cases. Spine J..

[4] Emile JF, Abla O, Fraitag S (2016). Revised classification of histiocytoses and neoplasms of the macrophage-dendritic cell lineages. Blood..

[5] Stalemark H, Laurencikas E, Karis J, Gavhed D, Fadeel B, Henter JI (2008). Incidence of Langerhans cell histiocytosis in children: a population-based study. Pediatr Blood Cancer..

[6] Bertram C, Madert J, Eggers C (2002). Eosinophilic granuloma of the cervical spine. Spine (Phila Pa 1976)..

[7] Huang WD, Yang XH, Wu ZP (2013). Langerhans cell histiocytosis of spine: a comparative study of clinical, imaging features, and diagnosis in children, adolescents, and adults. Spine J..

[8] Drevelegas A, Chourmouzi D, Boulogianni G, Sofroniadis I (2003). Imaging of primary bone tumors of the spine. Eur Radiol..

[9] Zhang X, Zhou J, Chai X (2018). The application of x-ray, computed tomography, and magnetic resonance imaging on 22 pediatric Langerhans cell histiocytosis patients with long bone involvement: A retrospective analysis. Medicine (Baltimore)..

[10] Rizzo FM, Cives M, Simone V, Silvestris F (2014). New insights into the molecular pathogenesis of langerhans cell histiocytosis. Oncologist..

[11] Abdel-Aziz M, Rashed M, Khalifa B, Talaat A, Nassar A (2014). Eosinophilic granuloma of the temporal bone in children. J Craniofac Surg..

[12] Inci M. F., Inci R., Ozkan F. (2013). Multidetector CT findings of calvarial eosinophilic granuloma. Case Reports.

[13] Arkader A, Glotzbecker M, Hosalkar HS, Dormans JP (2009). Primary musculoskeletal Langerhans cell histiocytosis in children: an analysis for a 3-decade period. J Pediatr Orthop..

[14] Hashmi MA, Haque N, Chatterjee A, Guha S (2012). Langerhans cell histiocytosis of long bones: MR imaging and complete follow up study. J Cancer Res Ther..

[15] Postini AM, Andreacchio A, Boffano M, Pagano M, Brach Del Prever A, Fagioli F (2012). Langerhans cell histiocytosis of bone in children: a long-term retrospective study. J Pediatr Orthop B..

[16] Chow TW, Leung WK, Cheng FWT (2017). Late outcomes in children with Langerhans cell histiocytosis. Arch Dis Child..

[17] Gao YJ, Su M, Tang JY, Pan C, Chen J (2018). Treatment Outcome of Children With Multisystem Langerhans Cell Histiocytosis: The Experience of a Single Children’s Hospital in Shanghai, China. J Pediatr Hematol Oncol..

[18] Asilsoy S, Yazici N, Demir S, Erbay A, Kocer E, Sarialioglu F (2017). A different cause for respiratory disorder in children: cases with pulmonary Langerhans cell histiocytosis. Clin Respir J..

[19] Yagci B, Varan A, Caglar M (2008). Langerhans cell histiocytosis: retrospective analysis of 217 cases in a single center. Pediatr Hematol Oncol..

[20] Saliba I, Sidani K, El Fata F, Arcand P, Quintal MC, Abela A (2008). Langerhans’ cell histiocytosis of the temporal bone in children. Int J Pediatr Otorhinolaryngol..

[21] Ribeiro KB, Degar B, Antoneli CB, Rollins B, Rodriguez-Galindo C (2015). Ethnicity, race, and socioeconomic status influence incidence of Langerhans cell histiocytosis. Pediatr Blood Cancer..

[22] Alston RD, Tatevossian RG, McNally RJ, Kelsey A, Birch JM, Eden TO (2007). Incidence and survival of childhood Langerhans cell histiocytosis in Northwest England from 1954 to 1998. Pediatr Blood Cancer..

[23] Muller J, Garami M, Hauser P (2006). Hungarian experience with Langerhans cell histiocytosis in childhood. Pediatr Hematol Oncol..

[24] Chen RL, Lin KS, Chang WH (2003). Childhood Langerhans cell histiocytosis increased during El Nino 1997-98: a report from the Taiwan Pediatric Oncology Group. Acta Paediatr Taiwan..

[25] Xu X, Han S, Jiang L (2018). Clinical features and treatment outcomes of Langerhans cell histiocytosis of the spine. Spine J..

[26] Wang D, Cui L, Li ZG (2018). Clinical research of pulmonary langerhans cell histiocytosis in children. Chin Med J (Engl)..

[27] Zhu H, Ma Y, Sun L, Zhang R, Lv L, Wang A (2019). Langerhans cell histiocytosis with lymph node involvement presenting as erythroderma. Acta Derm Venereol..

[28] Su Meng, Gao Yi-Jin, Pan Ci, Chen Jing, Tang Jing-Yan (2018). Outcome of children with Langerhans cell histiocytosis and single-system involvement: A retrospective study at a single center in Shanghai, China. Pediatric Hematology and Oncology.

