# Supplementary material for: Incidence and radiological pattern of eosinophilic granuloma: a retrospective study in a Chinese tertiary hospital
Source: J Orthop Surg Res. 2019 May 9;14:123. doi: 10.1186/s13018-019-1158-1 (PMC6507022; doi:10.1186/s13018-019-1158-1)
Supplement: Supplementary file 1 — Table S1. Basic information of 76 patients. (DOCX 29 kb) [file 13018_2019_1158_MOESM1_ESM.docx]

**Supplementary Table 1** Basic information of 76 patients.

| Patient | | Sex/Age | Smoking, year | Size, cm (long length) | Anatomical site |
| --- | --- | --- | --- | --- | --- |
| 1 | F/27 | | 7 | 2.0 | Frontal, temporal, parietal and occipital bone |
| 2 | F/7 | |  | 3.5 | Right femur |
| 3 | M/4 | |  | 10.0 | Right humerus |
| 4 | M/13 | |  | Puncture | Sacral vertebra |
| 5 | M/6 | |  | 3.0 | Frontal bone |
| 6 | M/10 | |  | 3.0 | Right occipital bone |
| 7 | M/3 | |  | 3.0 | Left femur |
| 8 | M/26 | |  | 4.0 | The 10th thoracic vertebra |
| 9 | M/5 | |  | 3.0 | Right radius |
| 10 | M/2 | |  | Puncture | Left ilium |
| 11 | F/8 | |  | 2.0 | Frontal bone |
| 12 | F/2 | |  | 3.0 | Frontal bone |
| 13 | F/3 | |  | 2.0 | Right femur |
| 14 | M/7 | |  | 6.0 | Frontal bone |
| 15 | F/2 | |  | 2.0 | Left humerus |
| 16 | M/15 | |  | 1.5 | Left clavicle |
| 17 | M/22 | |  | 2.0 | Left lacrimal gland |
| 18 | F/5 | |  | 3.0 | Right humerus |
| 19 | M/11 | |  | 10.0 | Right mandible |
| 20 | M/1 | |  | 6.0 | Left eighth rib |
| 21 | M/2 | |  | 1.5 | Left femur |
| 22 | M/5 | |  | 6.0 | Left ilium |
| 23 | M/54 | | 35 | 1.0 | Left ninth rib |
| 24 | M/16 | |  | 1.0 | Right femur |
| 25 | M/3 | |  | 2.5 | Right tibia |
| 26 | M/19 | |  | Puncture | Skin |
| 27 | M/22 | | 3 | 5.0 | Left third and fourth rib |
| 28 | M/22 | |  | 3.0 | Left neck, binaural |
| 29 | M/2 | |  | 5.0 | Left third and fourth rib |
| 30 | M/36 | | 10 | 3.0 | Left third rib |
| 31 | F/51 | |  | Puncture | Second lumbar vertebra |
| 32 | M/11 | |  | 1.5 | Right sciatic, acetabulum |
| 33 | M/1 | |  | 1.5 | Left femur |
| 34 | M/7 | |  | 3.0 | Right femur |
| 35 | M/9 | |  | 9.0 | Thoracic vertebral canal |
| 36 | F/52 | |  | 1.5 | Skin |
| 37 | M/20 | | 4 | 4.0 | Frontal bone |
| 38 | M/4 | |  | 3.0 | Sternum |
| 39 | M/58 | |  | 3.0 | Left fifth rib |
| 40 | M/33 | |  | 3.0 | Right third rib |
| 41 | M/50 | | 30 | 3.0 | Left clavicle |
| 42 | F/10 | |  | 5.0 | Left clavicle |
| 43 | M/47 | |  | 3.0 | Bilateral sacroiliac joint, sacrum, left ilium, left fifth rib |
| 44 | F/4 | |  | 3.0 | Right femur |
| 45 | M/2 | |  | 3.0 | Left femur |
| 46 | F/1 | |  | 5.0 | Left femur |
| 47 | M/1 | |  | Puncture | Neck, groin |
| 48 | F/9 | |  | 3.0 | Right tibia |
| 49 | M/31 | | 5 | 1.5 | Right pontocerebellar |
| 50 | M/24 | |  | 2.0 | Right sacroiliac joint |
| 51 | F/19 | |  | Puncture | Right pubis |
| 52 | M/10 | |  | 6.0 | Left eighth rib and diaphragm |
| 53 | F/24 | |  | 2.0 | Left parietal lobe |
| 54 | M/29 | |  | 5.0 | Right fourth rib |
| 55 | M/3 | |  | 2.0 | Bilateral orbit |
| 56 | M/1 | |  | 3.0 | Right orbit |
| 57 | M/23 | |  | 1.0 | The right third metacarpal |
| 58 | M/25 | | 8 | 4.0 | Left neck, left armpit |
| 59 | M/38 | | 20 | 5.0 | Right ninth rib |
| 60 | M/3 | |  | Puncture | Right orbit |
| 61 | M/20 | |  | Puncture | Right scapula |
| 62 | M/37 | |  | Puncture | Left neck, groin, abdominal cavity |
| 63 | F/54 | |  | 6.0 | Left seventh rib |
| 64 | M/5 | |  | 2.0 | Right femur |
| 65  66  67  68  69  70  71  72  73  74  75  76 | F/3  M/9  F/36  F/51  M/41  F/13  M/32  F/23  F/34  M/50  F/7  M/4 | | 20  10 | 2.0  3.0  1.5  3.0  Puncture  3.0  6.0  Puncture  Puncture  8.0  5.0  2.0 | Left ilium  Occipital bone  Left sciatic  Left tibia  Left parotid gland  Occipital bone and left parietal bone  Right tenth rib  Sacrum  Right clavicle  Right parotid gland  Right temporal bone  Left humerus |

***Note:** the location of the puncture or operation was mentioned only in the table.
