# Supplementary figures and images for: Incidence and radiological pattern of eosinophilic granuloma: a retrospective study in a Chinese tertiary hospital
Source: J Orthop Surg Res. 2019 May 9;14:123. doi: 10.1186/s13018-019-1158-1 (PMC6507022; doi:10.1186/s13018-019-1158-1)

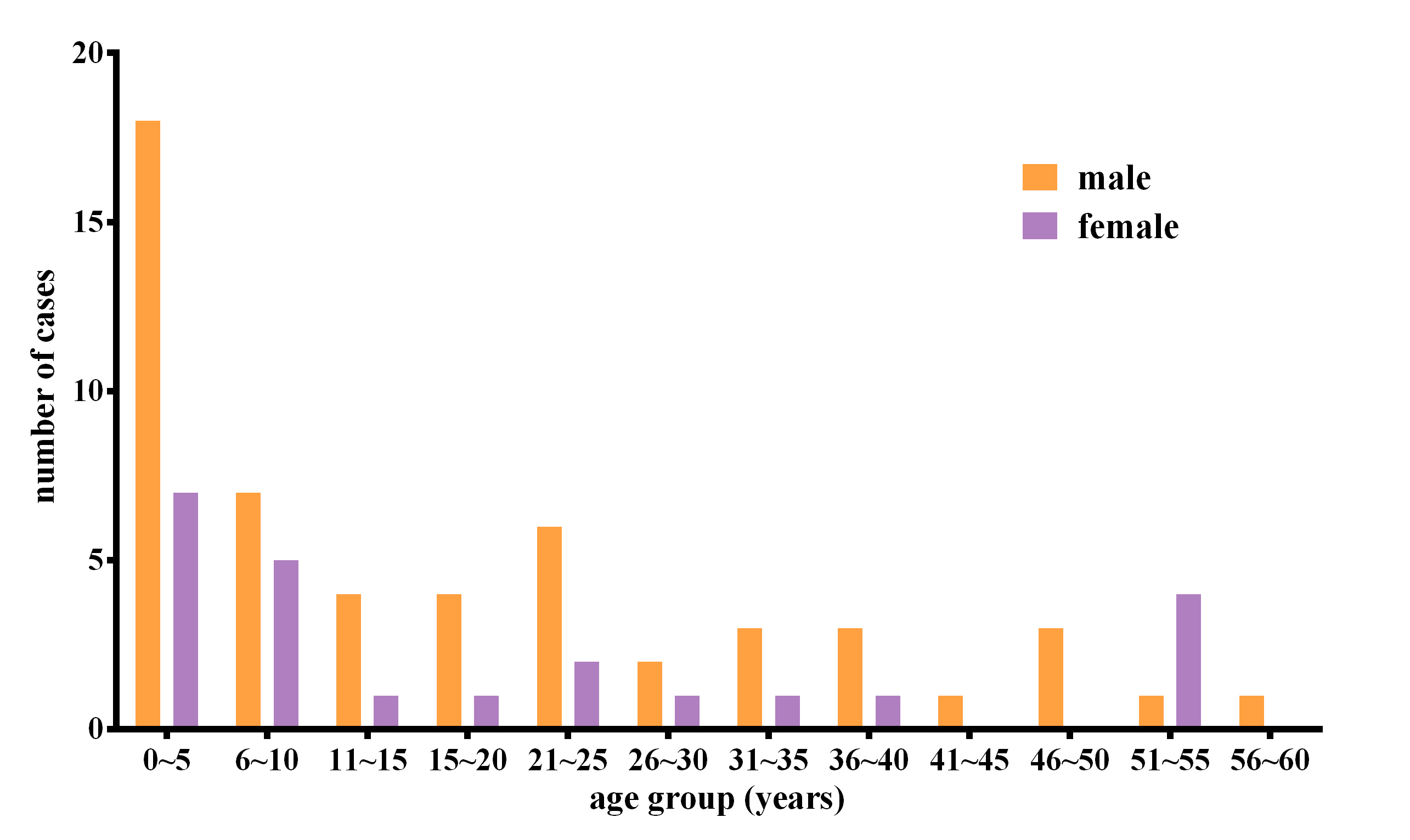

Supplement: Supplementary file 2 — Figure S1. Gender differences among different age groups. (JPG 293 kb) [file 13018_2019_1158_MOESM2_ESM.jpg]

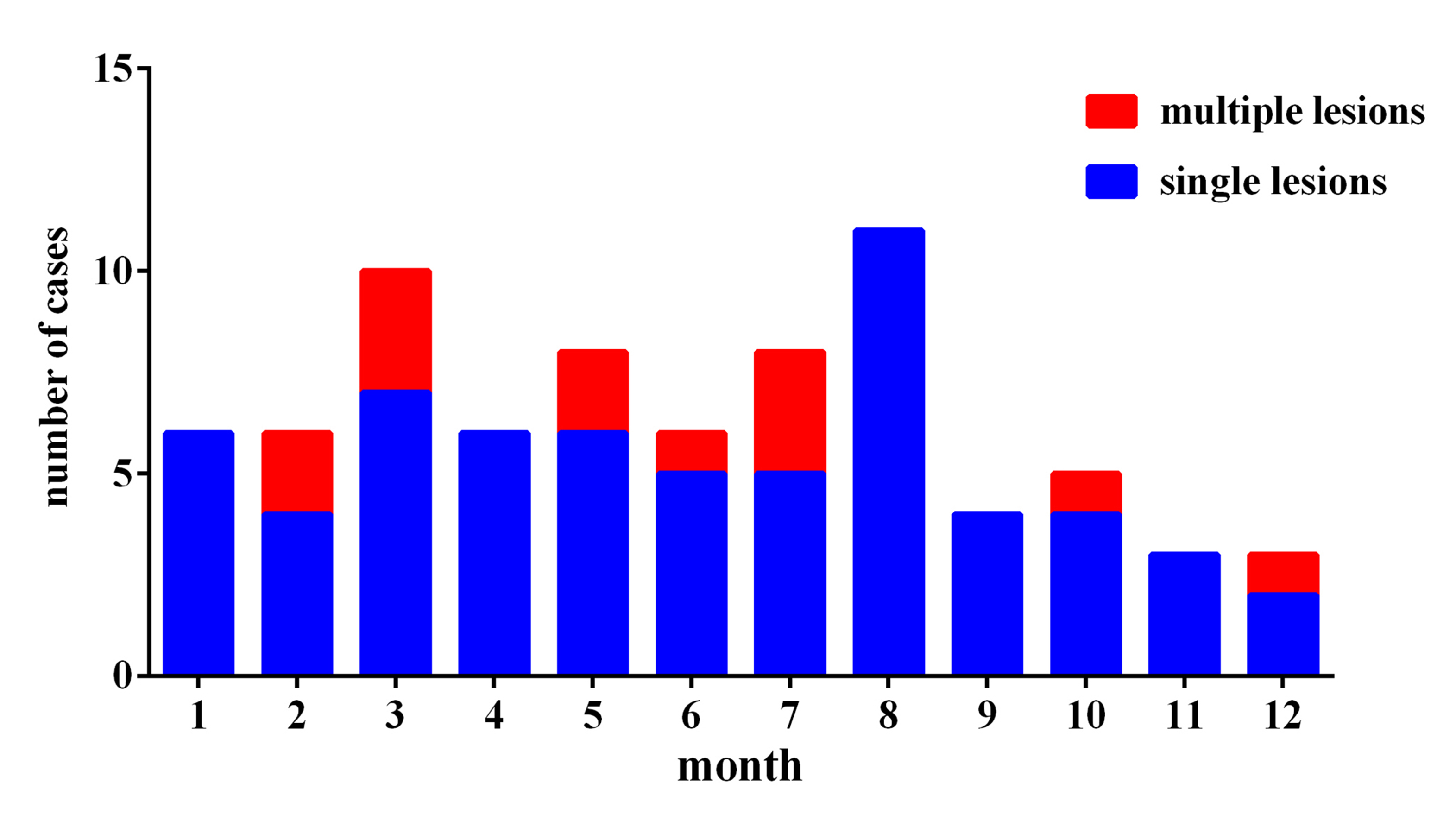

Supplement: Supplementary file 3 — Figure S2. Seasonal variation of EG. (JPG 317 kb) [file 13018_2019_1158_MOESM3_ESM.jpg]
