# Supplementary material for: Incidence and radiological pattern of eosinophilic granuloma: a retrospective study in a Chinese tertiary hospital
Source: J Orthop Surg Res. 2019 May 9;14:123. doi: 10.1186/s13018-019-1158-1 (PMC6507022; doi:10.1186/s13018-019-1158-1)
Supplement: Supplementary file 4 — Table S2. Pathological findings obtained by imaging diagnosed as “EG”. (DOCX 30 kb) [file 13018_2019_1158_MOESM4_ESM.docx]

**Supplementary Table 2** Pathological findings obtained by imaging diagnosed as “EG”

| Patient | Sex/Age | Anatomical location | X-ray(35)  13(37.1%) | CT(30)  5(16.7%) | MRI(36)  8(22.2%) | Pathologic diagnosis |
| --- | --- | --- | --- | --- | --- | --- |
| 1 | F/27 | Frontal, temporal, parietal and occipital bone |  |  |  | EG |
| 2 | F/7 | Right femur |  |  |  | EG |
| 3 | M/4 | Right humerus |  |  |  | EG |
| 4 | M/13 | Sacral vertebra |  |  |  | EG |
| 5 | M/6 | Frontal bone |  |  |  | EG |
| 6 | M/10 | Right occipital bone |  |  |  | EG |
| 7 | M/3 | Bilateral humerus, left ulna, left femur, right tibia, thoracic vertebra |  |  |  | EG |
| 8 | M/26 | The 10th thoracic vertebra |  |  |  | EG |
| 9 | M/5 | Right radius |  |  |  | EG |
| 10 | M/2 | Left ilium |  |  |  | EG |
| 11 | F/8 | Left ilium |  |  |  | EG |
| 12 | F/2 | Right ilium |  |  |  | EG |
| 13 | F/3 | Right femur |  |  |  | EG |
| 14 | M/6 | Right parietal |  |  |  | EG |
| 15 | F/2 | Left humerus |  |  |  | EG |
| 16 | M/14 | Left clavicle |  |  |  | EG |
| 17 | F/8 | Right temporal bone |  |  |  | EG |
| 18 | M/25 | Right parietal bone |  |  |  | EG |
| 19 | F/14 | Occipital and left parietal bone |  |  |  | EG |
| 20 | M/10 | Occipital bone |  |  |  | EG |
| 21 | M/5 | Left humerus, femur and right ulna, tibia |  |  |  | EG |
| 22 | M/1 | Left orbit |  |  |  | EG |
| 23 | M/11 | Right humerus |  |  |  | Non hodgkin lymphoma |
| 24 | M/16 | Right humerus |  |  |  | Osteosarcoma |
| 25 | M/15 | Right parietal bone |  |  |  | Epidermoid cyst |
| 26 | F/5 | Right femur |  |  |  | Enchondroma |
| 27 | F/13 | Right femur |  |  |  | Neuroblastoma |
| 28 | M/17 | Left elbow |  |  |  | Tuberculosis |
| 29 | M/6 | Right radius |  |  |  | Pyogenic osteomyelitis |
| 30 | M/21 | Sternum |  |  |  | Non hodgkin lymphoma |
| 31 | F/17 | Right acetabulum |  |  |  | Malignant mesenchymal tumor |
| 32 | M/15 | Left femur |  |  |  | Malignant mesenchymal tumor |
| 33 | M/2 | Frontal and parietal bone |  |  |  | Desmoid fibroma |
| 34 | F/32 | Right tibia |  |  |  | Aneurysmal bone cyst |
| 35 | M/13 | Left pubis |  |  |  | Ewing’s sarcoma |
| 36 | M/14 | Right parietal bone |  |  |  | Osteoma |
| 37 | M/2 | Left femur |  |  |  | Chronic granulomatous inflammation |
| 38 | F/9 | The third and fourth phalanx of left hand |  |  |  | Enchondroma |
| 39 | F/38 | Right ankle joint |  |  |  | Dendritic cell sarcoma |
| 40 | F/56 | Occipital bone |  |  |  | Anaplastic hemangioma |
| 41 | F/46 | Right tibia |  |  |  | Anaplastic large cell lymphoma |
| 42 | F/4 | Right femur |  |  |  | Chronic osteomyelitis |
| 43 | M/27 | Right humerus |  |  |  | Adenocarcinoma |
| 44 | F/47 | Right temporal bone |  |  |  | Osteoma |
| 45 | M/13 | Right femur |  |  |  | Fibrous dysplasia of bone |
| 46 | M/40 | Left temporal bone |  |  |  | Osteoma |
| 47 | M/19 | Right fifth rib |  |  |  | Fibrous dysplasia of bone |
| 48 | F/29 | Left eighth rib |  |  |  | Ossifying fibroma |
| 49 | M/18 | Right clavicle |  |  |  | Inflammatory granuloma |
| 50 | F/31 | Right ulna |  |  |  | Giant cell tumor of bone |
| 51 | M/36 | Occipital and left temporal parietal bone |  |  |  | Undifferentiated pleomorphic sarcoma |
| 52 | M/7 | Left femur |  |  |  | Osteoid osteoma |
| 53 | M/11 | Left tibia |  |  |  | Bone cyst |
| 54 | F/5 | Left femur |  |  |  | Fibrous dysplasia of bone |
| 55 | M/5 | Right femur |  |  |  | Small cell tumor |
| 56 | M/11 | Left orbit |  |  |  | Ossifying fibroma |
| 57 | F/56 | Occipital and bilateral parietal bone |  |  |  | Anaplastic hemangioma |
| 59 | F/20 | Right parietal bone |  |  |  | Dermoid cyst |
| 59 | F/23 | Twelfth thoracic vertebra |  |  |  | Giant cell tumor of bone |
| 60 | F/28 | Left clavicle |  |  |  | Bone tuberculosis |

√ pathologically confirmed EG;

X pathologically confirmed non-EG lesions.
